# Vigorous physical activity and physical function are strongly associated with better health in patients with long-standing radiographic axial spondyloarthritis

**DOI:** 10.1371/journal.pone.0360248

**Published:** 2026-10-13

**Authors:** Caroline Feldthusen, Magnus Hallström, Erik Hulander, Anna Deminger, Helena Forsblad-d’Elia

**Affiliations:** 1 Department of Health and Rehabilitation, Institute of Neuroscience and Physiology, Sahlgrenska Academy at University of Gothenburg, Gothenburg, Sweden; 2 Department of Occupational therapy and Physiotherapy, Region Västra Götaland, Sahlgrenska University Hospital, Gothenburg, Sweden; 3 Department of Rheumatology and Inflammation Research, Institute of Medicine, Sahlgrenska Academy at University of Gothenburg, Gothenburg, Sweden; 4 Department of Rheumatology, Region Västra Götaland, Sahlgrenska University Hospital, Gothenburg, Sweden; 5 Region Västra Götaland, Sahlgrenska University Hospital, Clinical Nutrition Unit, Gothenburg, Sweden; Prime Hospital LLC, UNITED ARAB EMIRATES

## Abstract

**Objectives:**

To evaluate the impact of physical function and level of physical activity on health status and health-related quality of life (HRQoL) in patients with long-standing radiographic axial spondyloarthritis (r-axSpA).

**Methods:**

In total, 141 patients with long-standing r-axSpA were included in this cross-sectional cohort study. Assessments included physical function (Bath Ankylosing Spondylitis Functional Index (BASFI)), spinal mobility (modified Bath Ankylosing Spondylitis Metrology Index (BASMI)), muscle function for leg strength (Timed Stands Test (TST)) and handgrip force (Grippit), amount of self-reported physical activity (vigorous physical activity, moderate physical activity, sedentary behavior), health status by ASAS Health Index (ASAS HI) and HRQoL with Short Form Survey Instrument 36-Item. Multivariable linear regression was used to analyze associations between health status, HRQoL, physical activity, muscle function, and physical function.

**Results:**

The mean (SD) age was 60 (12) years, diagnosis duration was 25 (10) years, and 57% were male. Physical function and vigorous physical activity were the strongest determinants of health status and HRQoL. The models explained approximately 50% of the variation in ASAS HI and the physical aspects of HRQoL and 21% of the mental aspects of HRQoL. Participants displayed good health status (62%) by ASAS HI ≤ 5, reached the recommended level of health-enhancing physical activity (77%), had normal muscle function (TST 82% and Grippit 91%), and demonstrated physical function (55%), and spinal mobility (45%) within reference values.

**Conclusion:**

This study highlights the importance of regular vigorous physical activity in older individuals living with long-standing r-axSpA since it was strongly associated with health status and HRQoL.

## Introduction

Radiographic axial spondyloarthritis (r-axSpA) is a chronic rheumatic inflammatory disease that results in long-lasting back pain and stiffness [1–3]. The disease is characterized by inflammation in the axial skeleton, enthesitis, and sometimes peripheral arthritis, as well as, to a varying degree, extra-musculoskeletal manifestations (EMMs): anterior uveitis, inflammatory bowel disease, and psoriasis. In addition to pain and stiffness, the impact on physical function and spinal mobility are crucial for patients with r-axSpA, as well as the impact on muscle function, especially muscle strength in the lower extremities [4]. Health-related outcomes and quality of life are negatively affected [5], and we recently found that both physical and mental health-related quality of life (HRQoL) were reduced in individuals with r-axSpA compared to controls from the general population [6]. Since the disease starts in early adulthood, its lifelong impact on physical function and health can be significant [2,7].

There is generally a decline in spinal mobility over time and a continuation of decrease even after 10 years of disease duration [8]. The continuous progress of structural damage in the spine is observed, according to the modified Stoke Ankylosing Spondylitis Spinal Score (mSASSS), especially in male patients [9,10].

Regular physical activity and exercise are a cornerstone in the management of r-axSpA [11]. Physical exercise programs have been shown to have a positive impact on physical function, spinal mobility, muscle function, and quality of life [12,13]. Vigorous high-intensity exercise has also shown effects on disease activity and inflammation [14–16]. However, these results are based on younger patient populations and less is known about the impact of vigorous physical activity on quality of life in older patients with long-standing disease. Furthermore, studies show that people with axSpA participate less in health-enhancing physical activity than population controls, especially vigorous physical activity [17,18]. Thus, little is known about the connection between different levels of physical activity, physical function, and quality of life in older patients with long-standing r-axSpA, and if there are differences between sexes. To develop and provide appropriate rehabilitation interventions for this group of patients, more data about this topic is needed.

The aim of this study was therefore to evaluate physical function, spinal mobility, muscle function, and the amount of different levels of physical activity to determine their influence on health status and HRQoL in patients with long-standing r-axSpA overall and stratified by sex. The secondary aims were to compare physical function, spinal mobility, muscle function, and physical activity in patients with r-axSpA compared to reference values from the general population to put the result into a broader context.

## Methods

### Study design

Cross-sectional cohort study.

### Participants

Patients in western Sweden were initially recruited to a cohort study in 2009 which has been previously described [19]. Inclusion criteria were diagnosis of r-axSpA according to the Modified New York Criteria (mNY) for ankylosing spondylitis (AS) [1] and age > 18 years. Exclusion criteria were psoriasis, inflammatory bowel disease, dementia, pregnancy, and difficulties in understanding Swedish language. The patients enrolled in 2009 were subsequently invited to follow-up examinations after 5-years in 2014 and 13-years in 2022–2023, as part of the Long-term Outcome Ankylosing Spondylitis (LOAS)-study. The present study focuses on the results from the 13-year follow-up in a cross-sectional manner. Out of originally 210 patients, 141 patients were included in the 13-year follow-up study from 05/01/2022 to 01/02/2023, see flowchart in (S1 Fig).

Written informed consent was obtained from all patients, and the study was approved by the Swedish Ethical Review Board (597–08, 2021–03484). The study was performed in compliance with the Declaration of Helsinki.

### Outcome measures

Analyzed data included socio-demographic factors (age, sex, disease duration, education (ranging from 1; 9-year compulsory school to 4; college/university), working status), medication, pain, and fatigue registered on Visual Analogue Scales (VAS) (0–100 mm) [20,21], and disease activity using Ankylosing Spondylitis Disease Activity Score (ASDAS) [22,23].

Health status was assessed with ASAS Health Index (ASAS HI) [24,25] ranging from 0 to 17, with a lower score indicating better health status, cut-off values ≤5 for good health, > 5 to <12 for moderate health, and ≥12 for poor health [26]. General health-related quality of life (HRQoL) was rated with the Short Form Survey Instrument 36-Item (SF36) [27] by the physical component summary (PCS) score and the mental component summary (MCS) score, which are standardized combined scores with a mean of 50 and a standard deviation of 10. A score above 50 represents better health than the general Swedish population [27].

Physical tests included spinal mobility measured with the modified Bath Ankylosing Spondylitis Metrology Index (BASMI) [28], range 0–10 (10 = worst) and muscle function measured using the Timed Stands Test (TST) [29] for leg strength, recording the time in seconds for 10 raises from a standard chair, and handgrip force measured using the dynamometer Grippit® (AB Detektor, Göteborg, Sweden) [30], recording the mean value during 10 s measured in Newton (N). For TST, a lower value represents greater leg strength, while for Grippit, a higher value represents greater handgrip force.

Self-reported questionnaires included physical function assessed with the Bath Ankylosing Spondylitis Functional Index (BASFI) [31], range 0–10 (10 = worst). Physical activity was an estimation of the mean physical activity of a regular week, reported for vigorous physical activity (i.e., regular physical exercise that makes you short of breath and sweaty like running, gymnastics, sports), from 0 (no time) to 5 (2 hours or more/week), moderate physical activity (daily activities like brisk walks, gardening, or bicycling) from 0 (no time) to 6 (5 hours or more/week), and sedentary behavior (apart from sleep, i.e., sitting) from 0 (never) to 6 (more than 15 hours/day) [32].

### Comparisons with reference values of healthy individuals

Measurements of physical function and physical tests of spinal mobility and muscle function were compared to reference values for a general population. For BASFI scores <1.5 in ages 30–50 years and <2.5 in ages above 50 were considered to correspond to a general population [33] and for BASMI age-matched reference values within or less than the third quartile (Q3) were considered representing normal spinal mobility [34]. TST and Grippit were compared to reference values pooled by age and sex [29,30]. Physical activity was evaluated in relation to the WHO guidelines for health-enhancing physical activity and sedentary behavior [35], i.e., 150–300 minutes moderate-intensity; or 75–150 minutes vigorous intensity; or a combination of these.

### Statistics

The study population was described using mean and standard deviation (SD) and/or median and quartile 1(Q1)/quartile 3(Q3) depending on the distribution of the variables. Categorical variables were described with number (n) and percentage (%). Non-parametric tests were used due to not normally distributed variables. Group comparisons were done using the Mann-Whitney U test and correlation analyses using Spearman’s rho. The strength of correlation was based on the predefined thresholds: negligible 0 to ±0.20, weak ± 0.21 to ±0.40, moderate ±0.41 to ±0.60, strong ±0.61 to ±0.80, and very strong ±0.81 to ±1.0 [36]. Regression models, using multivariable linear regression, were developed for the analysis of associations between the dependent variables ASAS- HI, SF36-PCS, and SF36-MCS and the independent variables age, BASFI, BASMI, TST, Grippit, vigorous physical activity, moderate physical activity, and sedentary behavior. The analyses were conducted in all patients and stratified by sex due to the differences in phenotype between sexes. Independent variables were chosen based on hypothetical reasoning and previous reports in the literature. Multicollinearity was examined by the Pearson correlation coefficient, r > 0.7 or r < −0.7, and the variance inflation factor. An analysis of residuals confirmed the assumptions of linearity by checking the residual scatterplots of each model, respectively. All significance tests were two-sided and conducted at the 5% significance level. Analyses were performed using the Statistical Package Software for the Social Sciences, SPSS version 29.0 (SPSS Inc., IBM, Chicago USA).

## Results

Descriptive data of the 141 participants with long-standing r-axSpA are presented in Table 1. The mean age (SD) of the population was 60 (12) years with a diagnostic duration of 25 (10) years and 57% were male. Fifty-six % were treated within specialist rheumatology care, the rest within primary care, and 42% were on disease-modifying anti-rheumatic drugs (DMARDs). According to ASDAS, 56% had inactive disease or low disease activity (<2.1). When comparing male and female participants, there was a significant difference between sexes in handgrip force (Grippit) (p < 0.001) and vigorous physical activity (p = 0.019), with male participants displaying greater handgrip force and higher amount of vigorous physical activity than females (Table 2).

**Table 1:** Characteristics of the patients with radiographic axial spondyloarthritis.

Variables n = 141	n (%)	Mean (SD) or Median (Q1;Q3)	Min–max
Age, years		60 (12)	30–84
Men, n	81 (57)		
Time since diagnosis, years†		25 (10)	14–52
Pain VAS, 0–100, score†		26.0 (9.3;50.0)	0–92.0
Fatigue VAS, 0–100, score†		27.5 (11.0;52.8)	0–94.0
ASDAS, score†		2.0 (1.3;2.6)	0.6–4.1
Specialist rheumatology clinic, n	79 (56)		
Medication*			
csDMARD	24 (17)		
bDMARD	49 (35)		
tsDMARD	1 (0.7)		
Daily NSAID	27 (19)		
**Work status**			
Fulltime	62 (44)		
Parttime	21 (15)		
Not working due to disability	13 (9)		
Unemployment	2 (1)		
Retirement	43 (31)		
**Education**			
compulsory school 9 years	15 (11)		
Upper secondary education	45 (32)		
Community college	9 (6)		
College/University	72 (51)		
**Physical function**			
BASFI, 0–10, score		1.9 (1.0;3.6)	0–8.2
BASMI, 0–10, score		2.8 (2.0;4.1)	0.6–7.4
TST, s†		15.2 (12.3;20.0)	8.1–74.2
Grippit, N		292 (220;392)	86–581
**Health and quality of life**			
ASAS HI, 0–17, score		4.0 (2;7)	0–17.0
SF36, PCS, score‡		42.7 (34.5;50.8)	17.6–59.1
SF36, MCS, score‡		49.4 (39.7;54.6)	9.5–62.5

n=number, s=seconds, N=Newton. †n=140, ‡n=139. VAS = Visual Analogue Scale, ASDAS = Axial Spondyloarthritis Disease Activity Score, DMARD = Disease-Modifying Anti-Rheumatic Drug, csDMARD = conventional synthetic DMARD, bDMARD = biologic DMARD, tsDMARD = targeted synthetic DMARD, NSAID = Non-Steroidal Anti-Inflammatory Drug, BASFI = Bath Ankylosing Spondylitis Functional Index, BASMI = Bath Ankylosing Spondylitis Metrology Index, TST = Timed Stands Test, ASAS HI = Assessment of SpondyloArthritis international Society Health Index, SF36 = Short Form Survey Instrument 36-Item, PCS = Physical Component Summary, MCS = Mental Component Summary. *15 patients were taking csDMARD in combination with bDMARD or tsDMARD.

**Table 2:** Comparisons between sexes in 141 patients with radiographic axial spondyloarthritis.

Variables	Males (n = 81)	Females (n = 60)	p
Age, years	58 (30–84)	61 (35–80)	0.41
Time since diagnosis, years	23 (14–52)	22 (14–52)	0.32
Pain VAS, 0–100, score	22 (0–82)	32.5 (0–92)	0.23
Fatigue VAS, 0–100, score	22 (0–86)	35 (0–94)	0.29
ASDAS, score	1.9 (0.6–4.1)	2.0 (0.6–3.8)	0.44
Physical function			
BASFI, 0–10, score	2.0 (0–8.2)	1.7 (0.04–8.2)	0.40
BASMI, 0–10, score	3.0 (0.6–7.4)	2.7 (0.6–6.8)	0.33
TST, s	15.7 (9.3–39.4)	14.8 (8.1–74.2)	0.21
Grippit, N	383 (106–581)	211 (86–361)	<0.001
**Physical activity**			
Vigorous PA, 0–5, score	3 (0–5)	2 (0–5)	0.019
Moderate PA, 0–6, score	4 (1–6)	4 (0–6)	0.61
Sedentary behaviour, 0–6, score	3 (1–6)	3 (1–6)	0.66
**Health and quality of life**			
ASAS HI, 0–17, score	4 (0–13)	5 (0–17)	0.13
SF36 PCS, score	43.9 (20.5–58.0)	42.2 (17.6–59.1)	0.35
SF36 MCS, score	51.0 (9.5–62.5)	47.8 (10.6–60.5)	0.055

Values are Median (Range), Mann-Whitney U test. n = number, s = seconds, N = Newton. VAS = Visual Analogue Scale, ASDAS = Axial Spondyloarthritis Disease Activity Score, BASFI = Bath Ankylosing Spondylitis Functional Index, BASMI = Bath Ankylosing Spondylitis Metrology Index, TST = Timed Stands Test, ASAS HI = Assessment of SpondyloArthritis international Society Health Index, SF36 = Short Form Survey Instrument 36-Item, PCS = Physical Component Summary, MCS = Mental Component Summary, PA = Physical Activity.

### Health status and HRQoL

Health status and HRQoL assessed with ASAS HI, SF36-PSC, and MSC are displayed in Table 1. For ASAS HI 62% of the participants displayed good health (a score <5). Only 4 participants (2.8%) displayed poor health status (a score >12). No statistically significant differences between sexes could be seen for either health outcome, but a non-significant trend was seen for SF36-MSC in which female participants reported poorer (lower scores <50) mental health than males (Table 2).

#### Univariate associations between physical function, spinal mobility, muscle function, level of physical activity, health status, and HRQoL.

Correlation analyses for health status and HRQoL are shown in Table 3. Physical function (BASFI) showed strong correlations with both ASAS-HI (r_s_ = 0.69) and SF36-PCS (r_s_ = −0.66). Spinal mobility (BASMI), muscle function (TST and Grippit), and vigorous physical activity showed weak to moderate correlations with both ASAS HI and SF36-PCS. Also, age correlated significantly with several measurements as shown in (S1 Table) including spinal mobility (BASMI) (r_s_ = 0.67), physical function (BASFI) (r_s_ = 0.44), and muscle strength (TST r_s_ = 0.55 and Grippit r_s_ = −0.49).

**Table 3:** Correlations between health status, health-related quality of life, physical function and physical activity, in 141 patients with radiographic axial spondyloarthritis.

Spearman’s rho (r_s_)	ASAS HI	SF36-PCS	SF36-MCS
Variables	r_s_ n=141	r_s_ n=139	r_s_ n=139
**Physical function**			
BASFI (Score)	0.69***	−0.66***	−0.23**
BASMI (Score)	0.40***	−0.40***	−0.01
TST (s)	0.47***	−0.47***	0.01
Grippit (N)	−0.37***	0.34***	0.10
**Physical activity**			
Vigorous PA	−0.40***	0.34***	0.23**
Moderate PA	−0.20*	0.21*	0.17*
Sedentary behaviour	−0.16	0.25**	−0.04

*p < 0.05, **p < 0.01, ***p < 0.001. n=number, s=seconds, N=Newton. ASAS HI = Assessment of SpondyloArthritis international Society Health Index, SF36 = Short Form Survey Instrument 36-Item, PCS = Physical Component Summary, MCS = Mental Component Summary, BASFI = Bath Ankylosing Spondylitis Functional Index, BASMI = Bath Ankylosing Spondylitis Metrology Index, TST = Timed Stands Test, PA = Physical Activity.

### Multivariable linear regression analyses with ASAS HI, SF36-PCS, and SF36-MCS as dependent variables

The models are presented in Table 4, overall and stratified by sex. The independent variables age, BASFI, BASMI, TST, Grippit, vigorous physical activity, moderate physical activity, and sedentary behavior were entered in all models.

**Table 4:** Linear multivariable regression models of health status and health related quality of life for patients with radiographic axial spondyloarthritis, total group and stratified by sex.

ASAS HI	Total group (N = 140)		Male (N = 80)		Female (N = 60)	
Adjusted R^2^	0.51		0.51		0.57	
	**B (95% CI)**	**P value**	**B (95% CI)**	**P value**	**B (95% CI)**	**P value**
*Intercept*	*7.00 (3.25;10.75)*	*<0.001*	*3.67 (−2.54;9.89)*	*0.24*	*11.57 (4.06;19.08)*	*0.003*
Age	−0.04 (−0.09;0.01)	0.13	−0.04 (−0.11;0.02)	0.19	−0.07 (−0.15;0.02)	0.11
BASFI	1.05 (0.76;1.34)	**<0.001**	0.96 (0.56;1.36)	**<0.001**	1.05 (0.63;1.47)	**<0.001**
BASMI	−0.05 (−0.45;0.36)	0.83	−0.09 (−0.57;0.40)	0.72	−0.05 (−0.50;1.02)	0.49
TST	0.02 (−0.04;0.09)	0.49	0.11 (0.02;0.22)	**0.045**	−0.05 (−0.13;0.04)	0.28
Grippit	−0.004 (−0.08;0.00)	0.06	0.003(−0.005;0.01)	0.47	−0.02(−0.04;-0.005)	**0.009**
Vigorous PA	−0.46 (−0.71;-0.21)	**<0.001**	−0.63 (−0.93;-0.33)	**<0.001**	−0.22 (−0.66;0.22)	0.32
Moderate PA	−0.05 (−0.31;0.22)	0.74	0.11(−0.24;0.47)	0.52	−0.17 (−0.57;0.24)	0.41
Sedentary behavior	−0.07 (−0.43;0.30)	0.73	−0.23 (−0.70;0.24)	0.33	0.28 (−0.29;0.84)	0.34
**SF36 PCS**	**Total group** (N = 138)		**Male** (N = 79)		**Female** (N = 59)	
Adjusted R^2^	0.48		0.50		0.49	
	**B (95% CI)**	**P value**	**B (95% CI)**	**P value**	**B (95% CI)**	**P value**
*Intercept*	*40.49 (29.78;51.19)*	*<0.001*	*42.49(23.67;61.31)*	*<0.001*	*41.71(20.73;62.70)*	*<0.001*
Age	0.04 (−0.10;0.19)	0.57	0.04 (−0.16;0.25)	0.66	−0.02 (−0.26;0.22)	0.87
BASFI	−2.41 (−3.23;-1.58)	**<0.001**	−3.06 (−4.28;-1.84)	**<0.001**	−1.47 (−2.65;-0.29)	**0.02**
BASMI	0.35 (−0.80;1.51)	0.55	1.13 (−0.33;2.59)	0.13	−0.46 (−2.58;1.65)	0.66
TST	−0.23 (−0.41;-0.05)	**0.01**	−0.29 (−0.62;0.04)	0.08	−0.17 (−0.41; 0.06)	0.15
Grippit	0.008 (−0.004;0.02)	0.17	0.01 (−0.009;0.04)	0.22	−0.007 (−0.05;0.03)	0.74
Vigorous PA	0.93 (0.21;1.65)	**0.01**	0.99 (0.08;1.91)	**0.03**	1.28 (0.06;2.50)	**0.04**
Moderate PA	0.31 (−0.45;1.07)	0.42	−0.52 (−1.60;0.57)	0.35	1.44 (0.32;2.56)	**0.01**
Sedentary behavior	0.73 (−0.32;1.77)	0.17	0.33 (−1.10;1.76)	0.65	0.72 (−0.87;2.31)	0.37
**SF36 MCS**	**Total group** (N = 138)		**Male** (N = 79)		**Female** (N = 59)	
Adjusted R^2^	0.21		0.08		0.35	
	**B (95% CI)**	**P value**	**B (95% CI)**	**P value**	**B (95% CI)**	**P value**
*Intercept*	*27.83 (11.90;43.76)*	*<0.001*	*42.21(12.65;71.76)*	*0.006*	*23.47(−5.95;52.88)*	*0.11*
Age	0.28 (0.06;0.50)	**0.01**	0.26 (−0.06;0.57)	0.11	0.37 (0.03,0.70)	**0.03**
BASFI	−2.87 (−4.10;-1.65)	**<0.001**	−2.46 (−4.38;-0.55)	**0.01**	−3.17 (−4.83;-1.52)	**<0.001**
BASMI	0.50 (−1.21;2.22)	0.56	0.32 (−1.97;2.62)	0.78	−0.62 (−3.58;2.35)	0.68
TST	0.16 (−0.11;0.43)	0.25	0.003 (−0.51;0.52)	0.99	0.14 (−0.20;0.47)	0.41
Grippit	0.008(−0.01;0.03)	0.36	−0.02 (−0.06;0.02)	0.31	0.03 (−0.03;0.09)	0.36
Vigorous PA	1.22 (0.15;2.28)	**0.03**	0.70 (−0.74;2.14)	0.33	1.77 (0.06;3.48)	**0.04**
Moderate PA	0.19 (−0.94;1.32)	0.74	0.64 (−1.07;2.35)	0.46	−0.58 (−2.16;0.99)	0.46
Sedentary behavior	−0.58 (−2.14;0.97)	0.46	−0.64 (−2.89;1.61)	0.57	−0.40 (−2.63;1.83)	0.72

Independent variables: Age, BASFI, BASMI, TST, Grippit, vigorous PA, moderate PA, sedentary behaviour. N = number, B = unstandardized beta coefficients, CI = confidence interval, R^2^ = coefficient of determination. ASAS HI = Assessment of SpondyloArthritis international Society Health Index, SF36 = Short Form Survey Instrument 36-Item, PCS = Physical Component Summary, MCS = Mental Component Summary, BASFI = Bath Ankylosing Spondylitis Functional Index, BASMI = Bath Ankylosing Spondylitis Metrology Index, TST = Timed Stands Test, PA = Physical Activity.

#### ASAS HI.

In the total group and for the male subgroup, better physical function and a higher amount of vigorous physical activity were associated with better health status. For males, better leg strength was also associated with better health status. In the female subgroup, better physical function and greater handgrip force were associated with better health status. The total variation in ASAS HI was explained by the models by 51% in the total group and 51% and 57% in males and females respectively.

#### SF36-PCS.

In the total group as well as both subgroups, better physical function and a higher amount of vigorous physical activity were associated with better physical health. In the total group, better leg strength and for the female subgroup, a higher amount of moderate physical activity were also associated with better physical health. The total variation in SF36-PCS was explained by the models by 48% in the total group and 50% and 49% in males and females respectively.

#### SF36-MCS.

In the total group and the female subgroup, better physical function, lower age, and a higher amount of vigorous physical activity were associated with better mental health. In the male subgroup, only better physical function was associated with better mental health. The total variation in SF36-MCS was explained by the models by 21% in the total group and 35% in females. For the male subgroup, the model only explained 8% of the variation in SF36-MCS.

### Comparison of physical function, spinal mobility, muscle function, and physical activity between patients with r-axSpA and reference values from the general population

Data on physical function, spinal mobility, and muscle function in the patients with r-axSpA are shown in Table 1. When adjusting the scores for age and comparing them to the general population, 55% of the participants scored physical function (BASFI) within the cutoff score for healthy controls [33] and 45% of the participants displayed spinal mobility (BASMI) within the normal range [34].

For muscle function stratified by age and sex, 82% of the participants performed within the normal range for leg strength (TST) [29], and 91% within the normal range for handgrip force (Grippit) [30].

The distribution of vigorous physical activity, moderate physical activity, and sedentary behavior is presented in Fig 1. Fifty-one % of the participants engaged in vigorous physical activity >1 hour/week. According to the WHO guidelines, 77% of the participants reached the recommendations for health-enhancing physical activity.

**Fig 1:**
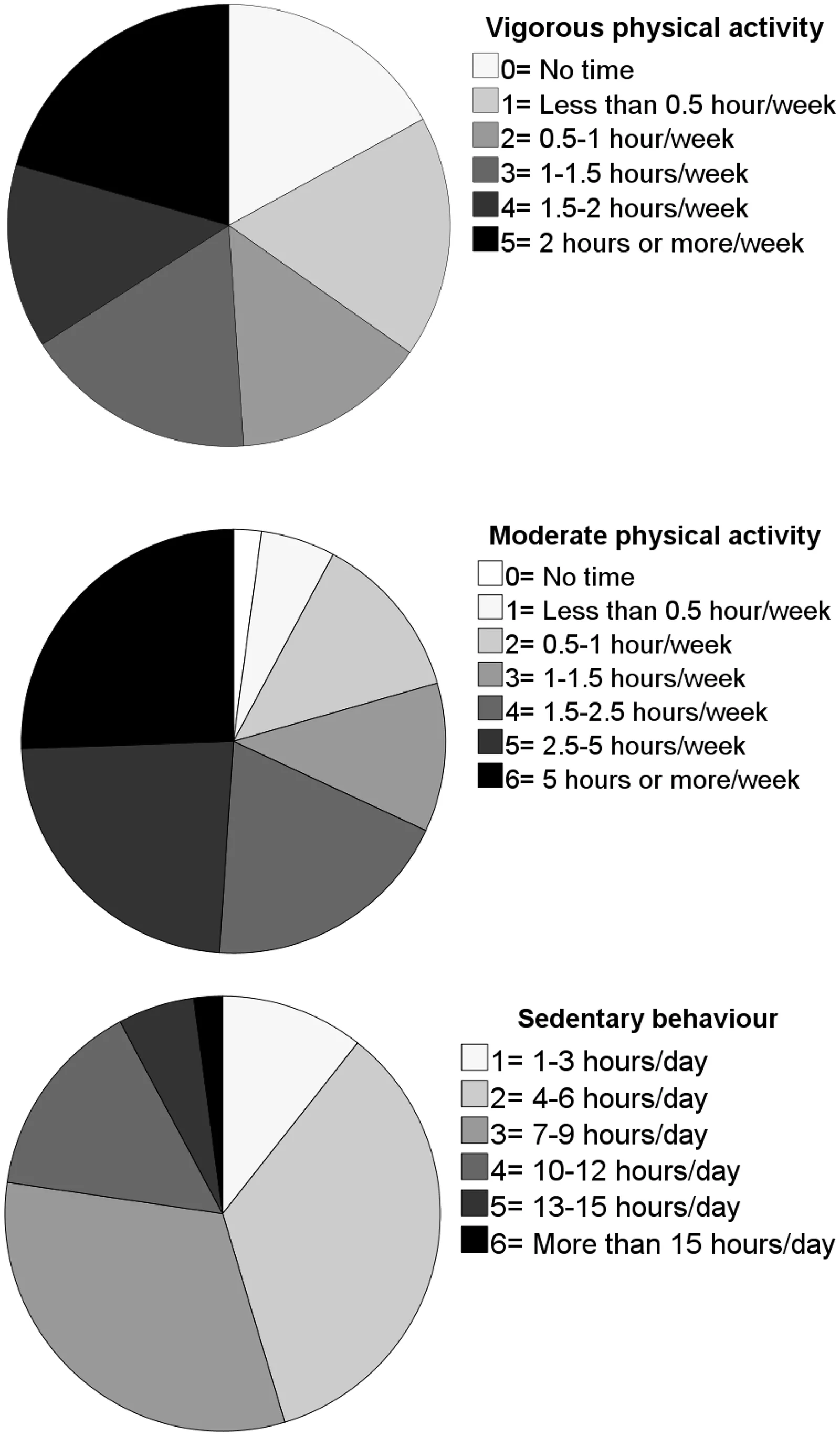
Physical activity behavior. The distribution of regular vigorous physical activity (running, gymnastics, sports, etc.), moderate physical activity (brisk walks, gardening, bicycling, etc.), and sedentary behavior apart from sleep (i.e., sitting).

## Discussion

The majority of the participants with long-standing r-axSpA displayed good health status assessed by ASAS HI. Physical function and vigorous physical activity were found to be strongly related to health status and the physical component of HRQoL. The models were able to explain approximately 50% of its variation while about 21% of the mental aspects of HRQoL in this study with older patients with r-axSpA.

Physical activity is first-line treatment in the management of axSpA [11] and the result of our study corresponds to the results of another recent study showing associations between physical activity at a moderate level or above and health status in younger patients with axSpA [37]. However, adherence to physical activity recommendations is suggested to be low in people with axSpA, with estimates varying from 17% to 71% [38]. In a general Swedish population in 2022 [39], 66% (age 30–84 years) were physically active at least 150 min/week on a moderate level, and in a previous Swedish questionnaire-based axSpA study, 68% reached this level [40]. In our population, 77% reached the level of health-enhancing physical activity, implying that they were more physically active than the general Swedish population [39] and also more physically active than previous axSpA populations [38,40]. We found that regular vigorous physical activity that makes you short of breath and sweaty seems to have a greater impact on health status than moderate intensity daily activities. This was seen especially among males who also reported engaging in more vigorous physical activity than the female patients. Research on vigorous high-intensity exercise has previously shown effects on both disease activity, inflammation, and physical function as well as fatigue, sleep, and general health in patients with r-axSpA [14,15] and a recent review concluded that aerobic exercise programs have an effect on C-reactive protein (CRP) and disease activity in r-axSpA [16]. However, the results of these studies were based on younger patients with axSpA compared with our older adults. Swinnen TW *et al.* reported that when compared to healthy individuals, time spent in light or moderate physical activity did not differ between patients with r-axSpA and healthy individuals, but patients with r-axSpA reported less vigorous physical activity [41]. Interviews with patients participating in high-intensity exercise have shown that this type of exercise is perceived as challenging for both body and mind, but rapid bodily effects resulted in strengthened faith in one’s own body and a changed attitude and motivation for exercise [42]. In our study, 51% of the participants engaged in vigorous physical activities >1hour/week compared to 32% exercising at vigorous intensity for ≥30 minutes 2–3 times/week, in the previous Swedish axSpA cohort [40].

In this study of mostly older patients with longstanding r-axSpA, approximately half of the participants displayed physical function and spinal mobility within the normal range, compared to age-matched reference values of a general population, and the vast majority had normal muscle function. Comparing our result to a recent 10-year follow-up study, the level of disease activity was similar but physical function by BASFI and health status by ASAS HI were better in our cohort despite higher age (60 years compared to 44 years) [43]. The associations between physical function and health status seen in our study correspond to the results of other recent studies consisting of patients about 15–20 years younger [44–46]. However, these studies also demonstrate associations with disease activity [37,44,45] and sex [44,45] which was not found in our older population. In line with our results, they found no associations between spinal mobility and health status [46]. However, comparison of disability and health status between different r-axSpA cohorts might introduce biases. The organization of healthcare differs between countries. Our study included patients who were followed both in specialized rheumatology care as well as in primary care. The patients in primary care are not treated with DMARDs and may represent a healthier group but might also be an undertreated group. Further, the disability profile of r-axSpA is evolving due to the biological era, which suggests decreased functional impairment over time [47]. In our study, we saw an association between age and a decline in physical function and spinal mobility which may be due to disease duration. However, such decline is also seen in a normal population, indicating that age itself is associated with decreasing mobility and physical function [33,34]. No differences in physical function and spinal mobility were seen between sexes, and normative reference values for BASFI and BASMI recommend adjustment to age but not sex [33,34]. However, male patients with r-axSpA often display worse BASMI values than females with r-axSpA [10] while BASFI is often found without a clear difference between the sexes [48]. Both BASFI and BASMI are composite scores based on several questions and measures respectively. Despite the absence of sex differences in composite scores, the score of individual questions/measures may differ between male and female patients [48].

Limitations of the study include the population sample, which needs to be considered. The participants were initially included in 2009, in a cohort that consisted of 210 patients. This implies that the remaining 141 participants in this study are a selected population whose lifestyle or health literacy may differ from the initial population. The participants represented both rheumatology clinics and primary care, which affects the ability to compare the results to other cohorts, but in turn broadens the generalizability of the results. This study also used self-reported questionnaires, which need to be regarded especially for the reporting of physical activity that may be both overestimated and underestimated [49]. Further, reporting of physical activity differs between studies, which may affect comparisons of physical activity across studies.

Although the majority of the participants in this study reported good health status and physical function, living with this chronic disease is virtually undescribed and could be an important area for future qualitative research. Also, no conclusions can be drawn about causal relationships from this report, which is why studies of causality are warranted, especially with a focus on older patients. High-intensity exercise has recently emerged as an effective treatment in patients with r-axSpA [14–16]. However, none of the studies include older patients. Our study demonstrates the ability to engage in vigorous physical activity also in older age, therefore, future RCTs evaluating the effects of vigorous physical exercise in older patients with r-axSpA are warranted to support recommendations for physical activity in this older population.

## Conclusion

Our results show that older individuals with r-axSpA have the ability to engage in vigorous physical activity, even in the later stages of disease, which is associated with good health outcomes. Approximately half of the participants displayed physical function and spinal mobility within normal range and the vast majority had normal muscle function.

## Supporting information

S1 Fig Flowchart. Flowchart of the inclusion in the Long-term Outcome Ankylosing Spondylitis (LOAS)-cohort in western Sweden.
(DOCX)

S1 Table Univariate associations. Correlations between age, physical function and physical activity in patients with radiographic axial spondylarthritis.
(DOCX)
