# Supplementary material for: Vigorous physical activity and physical function are strongly associated with better health in patients with long-standing radiographic axial spondyloarthritis
Source: PLoS One. 2026 Oct 13;21(10):e0360248. doi: 10.1371/journal.pone.0360248 (PMC13660928; doi:10.1371/journal.pone.0360248)
Supplement: Supplementary file 1 [file pone.0360248.s001.docx]

**Patients with radiographic axial spondyloarthritis**

**Patients included in the 2009 western Sweden cohort**

**N=210**

Patients invited to 13-years follow-up in 2022, The LOAS-study

N=180

**Patients included in the LOAS-study**

**N=141**

**Patients included in the 5-years follow-up 2014**

**N=173**

-Death n=4

-Declined participation n=12

-Cancelled or no response n=21

-Death n=16

-Previous drop-outs n=8

-Moved out of region n=6

-Death n=1

-Declined participation n=12

-Cancelled or no response n=24

-Excluded due to dementia n=2

**S1 Fig.** Flowchart of the inclusion of patients in the 2009 Western Sweden cohort and the 13-year follow-up of the Long-term Outcome Ankylosing Spondylitis (LOAS)-study.
