# Supplementary material for: Vigorous physical activity and physical function are strongly associated with better health in patients with long-standing radiographic axial spondyloarthritis
Source: PLoS One. 2026 Oct 13;21(10):e0360248. doi: 10.1371/journal.pone.0360248 (PMC13660928; doi:10.1371/journal.pone.0360248)
Supplement: Supplementary file 2 [file pone.0360248.s002.docx]

**S1 Table. Correlations between age, physical function and physical activity in patients with radiographic axial spondylarthritis**

| Spearman’s rho (r_s_) | Age  n=141  r_s_ | BASFI  n=141  r_s_ | BASMI  n=141  r_s_ | TST  n=140  r_s_ | Grippit  n=141  r_s_ | Vigorous PA  n=141  r_s_ | Moderate PA  n=141  r_s_ |
| --- | --- | --- | --- | --- | --- | --- | --- |
| BASFI (Score) | 0.44*** |  |  |  |  |  |  |
| BASMI (Score) | 0.67*** | 0.66*** |  |  |  |  |  |
| TST (s) | 0.55*** | 0.51*** | 0.60*** |  |  |  |  |
| Grippit (N) | -0.49*** | -0,27** | -0.34*** | -0.33*** |  |  |  |
| Vigorous PA | -0.05 | -0,21* | -0.10 | -0.16 | 0.21* |  |  |
| Moderate PA | -0.02 | -0.17* | -0.01 | -0.09 | 0.10 | 0.32*** |  |
| Sedentary behavior | -0.15 | -0.17* | -0.13 | -0.12 | 0.10 | 0.11 | 0.03 |

*p<0.05, **p<0.01, ***p<0.001

n=number, s=seconds, N=Newton.

BASFI=Bath Ankylosing Spondylitis Functional Index, BASMI=Bath Ankylosing Spondylitis Metrology Index, TST=Timed Stands Test, PA=Physical Activity.
