# Supplementary material for: Vigorous physical activity and physical function are strongly associated with better health in patients with long-standing radiographic axial spondyloarthritis
Source: PLoS One. 2026 Oct 13;21(10):e0360248. doi: 10.1371/journal.pone.0360248 (PMC13660928; doi:10.1371/journal.pone.0360248)
Supplement: Supplementary file 7 [file pone.0360248.s008.docx]

PONE-D-25-27651R2

Vigorous physical activity and physical function are strongly associated with better health in patients with long-standing radiographic axial spondyloarthritis.

PLOS One

**Response to reviewer**

Reviewer #3: Minor comments to the Author:
1. On page 3, under Introduction, please clarify preference of "(EMMs); anterior uveitis," or "(EMMs), anterior uveitis,".

*Response: Thank you for this comment. The wording of the sentence has been improved to the following sentence. ”The disease is characterized by inflammation in the axial skeleton, enthesitis, and sometimes peripheral arthritis, as well as, to a varying degree, extra-musculoskeletal manifestations (EMMs): anterior uveitis, inflammatory bowel disease, and psoriasis”.*

2. On page S1_Fig, please clarify preference of "un-bolded box" or "bolded box" for "Patients invited to 13-years follow-up in 2022, The LOAS-study N=180".

*Response: The bolded boxes describe how many patients that were included at baseline, 5-years follow -up and 12-13 years follow-up, respectively. The un-bolded box describes the recruitment process and the number of patients invited for follow-up, this to make the process transparent. However, that box does not describe the patients included in the actual data collection. Therefor we prefer to keep that box un-bolded.*
