# Supplementary material for: Vigorous physical activity and physical function are strongly associated with better health in patients with long-standing radiographic axial spondyloarthritis
Source: PLoS One. 2026 Oct 13;21(10):e0360248. doi: 10.1371/journal.pone.0360248 (PMC13660928; doi:10.1371/journal.pone.0360248)
Supplement: Supplementary file 8 [file pone.0360248.s003.docx]

Vigorous physical activity and physical function are strongly associated with better health in patients with long-standing radiographic axial spondyloarthritis (r-axSpA).

This cross-sectional cohort study (N=141), based on registry data obtained from a longitudinal (13-year follow-up) cohort study (N=210), The Long-term Outcome Ankylosing Spondylitis (LOAS)-study in western Sweden (Ref#19, 2012), aim to evaluate physical function, spinal mobility, muscle function, and physical activity in patients with r-axSpA compared to reference values from the general population.

This manuscript is well written, presented results of original research, performed analysis/statistics at a high standard, included study limitations, and provided a well-focused discussion with appropriate conclusion supported by the data. Although this manuscript is technically rigorous and meets the scientific and ethical standard for inclusion in PLOS One, its average level of importance would also be of interest for general rheumatology, physical therapy, or rehabilitation journals (e.g. Annals of Rheumatic Disease, where the authors had published their abstract entitled "Physical function and regular exercise is important for Health-related Quality of life in patients with radiographic axial Spondyloarthritis" in 2024).

Specific Comments to the Authors:

1. On page 6, Comparisons with reference values of health individuals, line 137, consider changing "for BASMI age-matched reference values within the third quartile (Q3)" to "for BASMI age-matched reference values within or less than the third quartile (Q3)" for clarity.

2. On page 9, Health status and HRQoL, line 198, consider changing "female participants reported poorer mental health than males (Table 2)" to "female participants reported poorer (lower scores <50) mental health than males (Table 2)".

3. On page 14, SF36-PCS, line 244-245, consider changing "In the total group, better leg strength and for the female subgroup, a higher amount of moderate physical activity were" to "In the total group (and male subgroup), better leg strength and for the female subgroup, a higher amount of moderate physical activity were" if that is correct.

4. On page 17, Discussion, line 307-308, please clarify why in the current cohort study vigorous physical activity is defined as >1 hour/week, whereas, in the original cohort study it is defined as ≥30 minutes 2-3 times/week. Are the criteria for defining vigorous physical activity for the two cohorts (N=141 vs N=210, respectively) same/comparable?

5. On page 23, Reference#19, used in reference to page 4, Participants, line 95-96, please clarify if it was Reference#19 or another article which used/defined the term "LOAS" (The Long-term Outcome Ankylosing Spondylitis study).
