# Supplementary material for: Vigorous physical activity and physical function are strongly associated with better health in patients with long-standing radiographic axial spondyloarthritis
Source: PLoS One. 2026 Oct 13;21(10):e0360248. doi: 10.1371/journal.pone.0360248 (PMC13660928; doi:10.1371/journal.pone.0360248)
Supplement: Supplementary file 9 [file pone.0360248.s005.docx]

Vigorous physical activity and physical function are strongly associated with better health in patients with long-standing radiographi8c axial spondyloarthritis.

This cross-sectional cohort study (N=141) re-enrolled in 2022, was based on registry data/enrollment obtained from a longitudinal cohort study (with 13-year follow-up) initially started in 2009 (N=210), The Long-term Outcome Ankylosing Spondylitis (LOAS)- study in western Sweden (Ref #19, 2012), aimed to evaluate physical function, spinal mobility, muscle function, and physical activity in patients with r-axSpA compared to reference values from the general population.

Comments to the Author:

1. On page 4, under Participants, please clarify whether the term "The Long-term Outcome Ankylosing Spondylitis (LOAS)-study was first used to describe the initial cohort enrolled in 2009 (Ref #19) or for the present study cohort re-enrolled in 2022. Similarly, please clarify appropriate time utilization of this term "LOAS" in the supplementary Figure 1. Reference 19 (published in 2012) never used the term "LOAS". Thus, may also want to add in (under References) the first peer-reviewed publication that actually used and defined the term "LOAS".

2. On page 8, under Results, please clarify in the text whether the percent of patients on disease-modifying anti-rheumatic drugs (DMARDs) is correctly at 42%, as according to Table 1 it stated 17% were on csDMARD, 35% were on bDMARD, and 0.7% were on tsDMARD, totaling to 52% (or 52.7%) were on DMARDs.

3. On page 14, under SF36-PCS (and Table 4 on page 12), please clarify the statement "In the total group, better leg strength... were also associated with better physical health." According to Table 4, under SF36 PCS, since the TST p value for both Male (p=0.08) and Female (p=0.15) were nonsignificant, it is not clear to this reviewer how then can be p value for the TST Total group (Male + Female) can mathematically somehow become significant for better physical health/leg strength muscle function. Please recheck the data and p values in Table 4.
