# Supplementary material for: Vigorous physical activity and physical function are strongly associated with better health in patients with long-standing radiographic axial spondyloarthritis
Source: PLoS One. 2026 Oct 13;21(10):e0360248. doi: 10.1371/journal.pone.0360248 (PMC13660928; doi:10.1371/journal.pone.0360248)
Supplement: Supplementary file 10 [file pone.0360248.s006.docx]

Response to Reviewers

Journal Requirements:

When submitting your revision, we need you to address these additional requirements.

1. Please ensure that your manuscript meets PLOS ONE's style requirements, including those for file naming. The PLOS ONE style templates can be found at

<https://journals.plos.org/plosone/s/file?id=wjVg/PLOSOne_formatting_sample_main_body.pdf> and

[https://journals.plos.org/plosone/s/file?id=ba62/PLOSOne_formatting_sample_title_authors_affiliations.pdf](https://track.editorialmanager.com/CL0/https:%2F%2Fjournals.plos.org%2Fplosone%2Fs%2Ffile%3Fid=ba62%2FPLOSOne_formatting_sample_title_authors_affiliations.pdf/1/010f019d6307d12b-83ecf51e-4cba-4663-b554-f3c162e5e886-000000/Z2fmU1yPjHcPrnbxPLfXdflHAqWj93QwLCdAcJllUIw=257)

 Response: We have checked the style templates and tried our best to meet the style requirements.

2. We note that the grant information you provided in the ‘Funding Information’ and ‘Financial Disclosure’ sections do not match.

When you resubmit, please ensure that you provide the correct grant numbers for the awards you received for your study in the ‘Funding Information’ section.

 Response: When resubmitting the manuscript, I can only find "Funding information". I have checked it and the information is correct. I cannot find and revise the "Financial Disclosure" section. How do I find it and make sure they correspond?

The funders had no role in study design, data collection and analysis, decision to publish, or preparation of the manuscript.

3. We note that you have indicated that there are restrictions to data sharing for this study. For studies involving human research participant data or other sensitive data, we encourage authors to share de-identified or anonymized data. However, when data cannot be publicly shared for ethical reasons, we allow authors to make their data sets available upon request. For information on unacceptable data access restrictions, please see <http://journals.plos.org/plosone/s/data-availability#loc-unacceptable-data-access-restrictions>.

Before we proceed with your manuscript, please address the following prompts:

a) If there are ethical or legal restrictions on sharing a de-identified data set, please explain them in detail (e.g., data contain potentially identifying or sensitive patient information, data are owned by a third-party organization, etc.) and who has imposed them (e.g., a Research Ethics Committee or Institutional Review Board, etc.). Please also provide contact information for a data access committee, ethics committee, or other institutional body to which data requests may be sent.

Response: The data contain sensitive personal information and because of the risk of re-identification, disclosure of data would require a secrecy assessment according to Swedish law.

A limited dataset with de-identified participant data that supports the main findings can be made available from the corresponding author upon reasonable request.

b) If there are no restrictions, please upload the minimal anonymized data set necessary to replicate your study findings to a stable, public repository and provide us with the relevant URLs, DOIs, or accession numbers. Please see <http://www.bmj.com/content/340/bmj.c181.long> for guidelines on how to de-identify and prepare clinical data for publication. For a list of recommended repositories, please see <https://journals.plos.org/plosone/s/recommended-repositories>. You also have the option of uploading the data as Supporting Information files, but we would recommend depositing data directly to a data repository if possible.

Please update your Data Availability statement in the submission form accordingly.

Response: We have updated this statement according to the response above.

4. If the reviewer comments include a recommendation to cite specific previously published works, please review and evaluate these publications to determine whether they are relevant and should be cited. There is no requirement to cite these works unless the editor has indicated otherwise.

Response: No requirements to cite specific works have been recommended by the reviewers

Reviewers' comments:

Reviewer's Responses to Questions

**Comments to the Author**

1. Is the manuscript technically sound, and do the data support the conclusions?

The manuscript must describe a technically sound piece of scientific research with data that supports the conclusions. Experiments must have been conducted rigorously, with appropriate controls, replication, and sample sizes. The conclusions must be drawn appropriately based on the data presented.

Reviewer #1: Yes

Reviewer #2: Yes

Reviewer #3: Yes

2. Has the statistical analysis been performed appropriately and rigorously?

Reviewer #1: Yes

Reviewer #2: Yes

Reviewer #3: Yes

3. Have the authors made all data underlying the findings in their manuscript fully available?

The [PLOS Data policy](http://track.editorialmanager.com/CL0/http:%2F%2Fwww.plosone.org%2Fstatic%2Fpolicies.action%23sharing/1/010f019d6307d12b-83ecf51e-4cba-4663-b554-f3c162e5e886-000000/IyXYF2yAWBQEJP8d7Sx5Euzm4EODc_h8LDk0r7pg-yQ=257) requires authors to make all data underlying the findings described in their manuscript fully available without restriction, with rare exception (please refer to the Data Availability Statement in the manuscript PDF file). The data should be provided as part of the manuscript or its supporting information, or deposited to a public repository. For example, in addition to summary statistics, the data points behind means, medians and variance measures should be available. If there are restrictions on publicly sharing data—e.g. participant privacy or use of data from a third party—those must be specified.

Reviewer #1: Yes

Reviewer #2: Yes

Reviewer #3: Yes

4. Is the manuscript presented in an intelligible fashion and written in standard English?

PLOS ONE does not copyedit accepted manuscripts, so the language in submitted articles must be clear, correct, and unambiguous. Any typographical or grammatical errors should be corrected at revision, so please note any specific errors here.

Reviewer #1: Yes

Reviewer #2: Yes

Reviewer #3: Yes

5. Review Comments to the Author

Please use the space provided to explain your answers to the questions above. You may also include additional comments for the author, including concerns about dual publication, research ethics, or publication ethics. (Please upload your review as an attachment if it exceeds 20,000 characters)

Reviewer #1: This article assesses relationships between health status, HRQol and physical activity in relatively elderly patients with e-axSpA using a cross-sectional dataset.
This study revealed that health status and physical activity are closely correlated even in long-standing axial SpA patients.
Several points need to be clarified

1) Influence of lifestyle, daily physical activity, working status, economic status, educational background, etc., so-called social background.

Response: We agree that social background also may influence health status. The questionnaire about physical activity that the patients answered was an estimation of the mean physical activities during a regular week. This clarification has now been added in the manuscript in the description of the questionnaire. We therefore have no data on daily physical activity. Neither do we have any detailed information on the patients’ economic status. We do have information regarding working status and educational background which has been added to table 1.

Since the patient group was rather old (mean age 60 years) and 31% of the patients were retired due to old age, the ability to make reliable statistical assumptions regarding the impact of working status is compromised. Though, we have now, added a variable about working status and retirement in table 1 to make this transparent.

Regarding educational status there were significant but weak correlations between educational status and both ASAS HI (r_s_= -0.250, p<0.001) and SF36-PCS (r_s_= 0.228, p<0.001). But educational status did not affect any of the models when entered in the regression model for the total group.

We found no significant difference in work status or educational status between sexes.

2) Medication

Response: Use of medication is presented in table 1. There was a significant correlation between daily use of NSAID and SF36-PCS (r_s_= -0.203, p<0.05), however negligible. No other significant correlations were found between medication and health status. Use of medication did not affect any of the models when entered in the regression model for the total group.

3) If these factors affect health status, a statistical correction is needed.

Response: We found no influence on health status regarding use of medication or educational status, thus no correction for medication or educational status was needed.

As approximately one third of the patients was retired due to old age and 75% of the rest of the patients were working full time, working status was not considered a reliable factor for the analysis but is presented in table 1 to describe the population.

Reviewer #2: This is a well-conducted and clearly written cross-sectional study based on a long-term r-axSpA cohort. The methodology is sound, with appropriate use of validated instruments (BASFI, BASMI, ASAS-HI, SF-36) and multivariable regression analyses. The handling of non-parametric data, assessment of collinearity, and model validation are all appropriate. The Discussion adequately addresses the limitations regarding potential selection bias and causal inference. Overall, the statistical and methodological quality is sufficient, and the findings are consistent and clinically meaningful.

Response: Thank you for your positive comments.

Reviewer #3: Vigorous physical activity and physical function are strongly associated with better health in patients with long-standing radiographic axial spondyloarthritis (r-axSpA).

This cross-sectional cohort study (N=141), based on registry data obtained from a longitudinal (13-year follow-up) cohort study (N=210), The Long-term Outcome Ankylosing Spondylitis (LOAS)-study in western Sweden (Ref#19, 2012), aim to evaluate physical function, spinal mobility, muscle function, and physical activity in patients with r-axSpA compared to reference values from the general population.

This manuscript is well written, presented results of original research, performed analysis/statistics at a high standard, included study limitations, and provided a well-focused discussion with appropriate conclusion supported by the data. Although this manuscript is technically rigorous and meets the scientific and ethical standard for inclusion in PLOS One, its average level of importance would also be of interest for general rheumatology, physical therapy, or rehabilitation journals (e.g. Annals of Rheumatic Disease, where the authors had published their abstract entitled "Physical function and regular exercise is important for Health-related Quality of life in patients with radiographic axial Spondyloarthritis" in 2024).

Response: Thank you for the positive comments!

Specific Comments to the Authors:
1. On page 6, Comparisons with reference values of health individuals, line 137, consider changing "for BASMI age-matched reference values within the third quartile (Q3)" to "for BASMI age-matched reference values within or less than the third quartile (Q3)" for clarity.

Response: Thank you for this suggestion for clarity. We have changed according to your suggestion.

2. On page 9, Health status and HRQoL, line 198, consider changing "female participants reported poorer mental health than males (Table 2)" to "female participants reported poorer (lower scores <50) mental health than males (Table 2)".

Response: Thank you for this suggestion for clarity. We have changed according to your suggestion.

3. On page 14, SF36-PCS, line 244-245, consider changing "In the total group, better leg strength and for the female subgroup, a higher amount of moderate physical activity were" to "In the total group (and male subgroup), better leg strength and for the female subgroup, a higher amount of moderate physical activity were" if that is correct.

Response: Thank you for being observant. However, for SF36-PCS the p-value for leg strength (TST) for the male subgroup was 0.08. Our cut off was p<0.05, and even if 0.08 is borderline, we chose to consider leg strength as not significant in the male subgroup.

4. On page 17, Discussion, line 307-308, please clarify why in the current cohort study vigorous physical activity is defined as >1 hour/week, whereas, in the original cohort study it is defined as >30 minutes 2-3 times/week. Are the criteria for defining vigorous physical activity for the two cohorts (N=141 vs N=210, respectively) same/comparable?

Response: The two studies used different questionnaires with different response options for physical activity. That is the reason for the different definitions of vigorous physical activity in the different cohort studies. However, we find these definitions comparable.

5. On page 23, Reference#19, used in reference to page 4, Participants, line 95-96, please clarify if it was Reference#19 or another article which used/defined the term "LOAS" (The Long-term Outcome Ankylosing Spondylitis study).

Response: Yes, reference #19 is correct as it is the first published reference for this cohort. However, the term LOAS is not used in that manuscript since it reports baseline results and no follow-up data had yet been collected
