# Supplementary material for: Vigorous physical activity and physical function are strongly associated with better health in patients with long-standing radiographic axial spondyloarthritis
Source: PLoS One. 2026 Oct 13;21(10):e0360248. doi: 10.1371/journal.pone.0360248 (PMC13660928; doi:10.1371/journal.pone.0360248)
Supplement: Supplementary file 11 [file pone.0360248.s007.docx]

Response to reviewers

PONE-D-25-27651R1

Vigorous physical activity and physical function are strongly associated with better health in patients with long-standing radiographic axial spondyloarthritis.

Journal Requirements:

1. If the reviewer comments include a recommendation to cite specific previously published works, please review and evaluate these publications to determine whether they are relevant and should be cited. There is no requirement to cite these works unless the editor has indicated otherwise.

2. Please review your reference list to ensure that it is complete and correct. If you have cited papers that have been retracted, please include the rationale for doing so in the manuscript text, or remove these references and replace them with relevant current references. Any changes to the reference list should be mentioned in the rebuttal letter that accompanies your revised manuscript. If you need to cite a retracted article, indicate the article’s retracted status in the References list and also include a citation and full reference for the retraction notice.
Response: The reference list has been checked, and no references have been retracted.

There is an erratum for ref 3; Rudwaleit M et al 2009: The correction provides conflict of interest statements of individual authors which were not provided to the paper.

There is also an error published regarding ref 40; Haglund E et al 2012. The error is in a table. The authors state that the error does not affect the conclusion of the article.

[Note: HTML markup is below. Please do not edit.]

Reviewers' comments:

Reviewer's Responses to Questions

**Comments to the Author**

1. If the authors have adequately addressed your comments raised in a previous round of review and you feel that this manuscript is now acceptable for publication, you may indicate that here to bypass the “Comments to the Author” section, enter your conflict of interest statement in the “Confidential to Editor” section, and submit your "Accept" recommendation.

Reviewer #1: All comments have been addressed

Reviewer #3: (No Response)

2. Is the manuscript technically sound, and do the data support the conclusions?

The manuscript must describe a technically sound piece of scientific research with data that supports the conclusions. Experiments must have been conducted rigorously, with appropriate controls, replication, and sample sizes. The conclusions must be drawn appropriately based on the data presented.

Reviewer #1: Yes

Reviewer #3: Yes

3. Has the statistical analysis been performed appropriately and rigorously?

Reviewer #1: Yes

Reviewer #3: Yes

4. Have the authors made all data underlying the findings in their manuscript fully available?

The [PLOS Data policy](http://track.editorialmanager.com/CL0/http:%2F%2Fwww.plosone.org%2Fstatic%2Fpolicies.action%23sharing/1/010f019f823c5bbc-9beeb6f5-1d2c-4b79-85a3-8cf4d11affdd-000000/aenHQOkCQBok-NUseAOe_OAeRh7A0x7-CiY_i-Tq300=258) requires authors to make all data underlying the findings described in their manuscript fully available without restriction, with rare exception (please refer to the Data Availability Statement in the manuscript PDF file). The data should be provided as part of the manuscript or its supporting information, or deposited to a public repository. For example, in addition to summary statistics, the data points behind means, medians and variance measures should be available. If there are restrictions on publicly sharing data—e.g. participant privacy or use of data from a third party—those must be specified.

Reviewer #1: Yes

Reviewer #3: Yes

5. Is the manuscript presented in an intelligible fashion and written in standard English?

PLOS ONE does not copyedit accepted manuscripts, so the language in submitted articles must be clear, correct, and unambiguous. Any typographical or grammatical errors should be corrected at revision, so please note any specific errors here.

Reviewer #1: Yes

Reviewer #3: Yes

6. Review Comments to the Author

Please use the space provided to explain your answers to the questions above. You may also include additional comments for the author, including concerns about dual publication, research ethics, or publication ethics. (Please upload your review as an attachment if it exceeds 20,000 characters)

Reviewer #1: Dear authors,
Thank you for your appropriate response.
You have a guarantee of good quality, enough to publish this article.

Response: Thank you for this positive response

Reviewer #3: Vigorous physical activity and physical function are strongly associated with better health in patients with long-standing radiographi8c axial spondyloarthritis.

This cross-sectional cohort study (N=141) re-enrolled in 2022, was based on registry data/enrollment obtained from a longitudinal cohort study (with 13-year follow-up) initially started in 2009 (N=210), The Long-term Outcome Ankylosing Spondylitis (LOAS)- study in western Sweden (Ref #19, 2012), aimed to evaluate physical function, spinal mobility, muscle function, and physical activity in patients with r-axSpA compared to reference values from the general population.

Comments to the Author:
1. On page 4, under Participants, please clarify whether the term "The Long-term Outcome Ankylosing Spondylitis (LOAS)-study was first used to describe the initial cohort enrolled in 2009 (Ref #19) or for the present study cohort re-enrolled in 2022. Similarly, please clarify appropriate time utilization of this term "LOAS" in the supplementary Figure 1. Reference 19 (published in 2012) never used the term "LOAS". Thus, may also want to add in (under References) the first peer-reviewed publication that actually used and defined the term "LOAS".

Response: Thank you for pointing out this unclarity. It is true that the term LOAS-study was not used to describe the initial cohort but the 13-year follow-up cohort. We have clarified this in page 4 which now states:

“Patients in western Sweden were initially recruited to a cohort study in 2009 which has been previously described [19]. Inclusion criteria were diagnosis of r-axSpA according to the Modified New York Criteria (mNY) for ankylosing spondylitis (AS) [1] and age >18 years. Exclusion criteria were psoriasis, inflammatory bowel disease, dementia, pregnancy, and difficulties in understanding Swedish language. The patients enrolled in 2009 were subsequently invited to follow-up examinations after 5-years in 2014 and after 13-years in 2022-2023. The latter as part of the Long-term Outcome Ankylosing Spondylitis (LOAS)-study (ref PMID: 40660310). The present study focuses on the results from the 13-year follow-up in a cross-sectional manner. Out of originally 210 patients, 141 patients were included in the 13-year follow-up study from 05/01/2022 to 01/02/2023, see flowchart in supplementary Figure 1 (S1 Fig). “

We have added a reference that first defined the LOAS-study and revised the supplementary flowchart figure (S1 Fig) accordingly.

2. On page 8, under Results, please clarify in the text whether the percent of patients on disease-modifying anti-rheumatic drugs (DMARDs) is correctly at 42%, as according to Table 1 it stated 17% were on csDMARD, 35% were on bDMARD, and 0.7% were on tsDMARD, totaling to 52% (or 52.7%) were on DMARDs.

Response: The reason for this is that 15 patients were taking csDMARD in combination with bDMARD or tsDMARD. That is why the total score do not match. To clarify we have added this information as a footnote to the table.

3. On page 14, under SF36-PCS (and Table 4 on page 12), please clarify the statement "In the total group, better leg strength... were also associated with better physical health." According to Table 4, under SF36 PCS, since the TST p value for both Male (p=0.08) and Female (p=0.15) were nonsignificant, it is not clear to this reviewer how then can be p value for the TST Total group (Male + Female) can mathematically somehow become significant for better physical health/leg strength muscle function. Please recheck the data and p values in Table 4.

Response: Thank you for being observant. We have rechecked the data and all values are correct. The reason for the p value being significant for the total group and not the subgroups is the larger n which results in significance being achieved.
