# Supplementary material for: Vigorous physical activity and physical function are strongly associated with better health in patients with long-standing radiographic axial spondyloarthritis
Source: PLoS One. 2026 Oct 13;21(10):e0360248. doi: 10.1371/journal.pone.0360248 (PMC13660928; doi:10.1371/journal.pone.0360248)
Supplement: Peer review history

# Peer review: Decision Letter 0

**DOI:** 10.1371/journal.pone.0360248.r001

**Authors:** Wesam Gouda

6 Apr 2026

Dear Dr. Feldthusen,

Thank you for submitting your manuscript to PLOS ONE. After careful consideration, we feel that it has merit but does not fully meet PLOS ONE’s publication criteria as it currently stands. Therefore, we invite you to submit a revised version of the manuscript that addresses the points raised during the review process.

Please submit your revised manuscript by May 21 2026 11:59PM. If you will need more time than this to complete your revisions, please reply to this message or contact the journal office at plosone@plos.org. When you're ready to submit your revision, log on to https://www.editorialmanager.com/pone/ and select the 'Submissions Needing Revision' folder to locate your manuscript file.

- A letter that responds to each point raised by the academic editor and reviewer(s). You should upload this letter as a separate file labeled 'Response to Reviewers'.
- A marked-up copy of your manuscript that highlights changes made to the original version. You should upload this as a separate file labeled 'Revised Manuscript with Track Changes'.
- An unmarked version of your revised paper without tracked changes. You should upload this as a separate file labeled 'Manuscript'.

If you would like to make changes to your financial disclosure, please include your updated statement in your cover letter. Guidelines for resubmitting your figure files are available below the reviewer comments at the end of this letter.

If applicable, we recommend that you deposit your laboratory protocols in protocols.io to enhance the reproducibility of your results. Protocols.io assigns your protocol its own identifier (DOI) so that it can be cited independently in the future. For instructions see: https://journals.plos.org/plosone/s/submission-guidelines#loc-laboratory-protocols. Additionally, PLOS ONE offers an option for publishing peer-reviewed Lab Protocol articles, which describe protocols hosted on protocols.io. Read more information on sharing protocols at https://plos.org/protocols?utm_medium=editorial-email&utm_source=authorletters&utm_campaign=protocols.

As the corresponding author, your ORCID iD is verified in the submission system and will appear in the published article. PLOS supports the use of ORCID, and we encourage all coauthors to register for an ORCID iD and use it as well. Please encourage your coauthors to verify their ORCID iD within the submission system before final acceptance, as unverified ORCID iDs will not appear in the published article. *Only*  the individual author can complete the verification step; PLOS staff *cannot*  verify ORCID iDs on behalf of authors.

We look forward to receiving your revised manuscript.

Kind regards,

Wesam Gouda, MD,PhD

Academic Editor

PLOS One

Journal Requirements:

When submitting your revision, we need you to address these additional requirements.

1. Please ensure that your manuscript meets PLOS ONE's style requirements, including those for file naming. The PLOS ONE style templates can be found at

https://journals.plos.org/plosone/s/file?id=wjVg/PLOSOne_formatting_sample_main_body.pdf and

https://journals.plos.org/plosone/s/file?id=ba62/PLOSOne_formatting_sample_title_authors_affiliations.pdf

2. We note that the grant information you provided in the ‘Funding Information’ and ‘Financial Disclosure’ sections do not match.

When you resubmit, please ensure that you provide the correct grant numbers for the awards you received for your study in the ‘Funding Information’ section.

3. We note that you have indicated that there are restrictions to data sharing for this study. For studies involving human research participant data or other sensitive data, we encourage authors to share de-identified or anonymized data. However, when data cannot be publicly shared for ethical reasons, we allow authors to make their data sets available upon request. For information on unacceptable data access restrictions, please see http://journals.plos.org/plosone/s/data-availability#loc-unacceptable-data-access-restrictions.

Before we proceed with your manuscript, please address the following prompts:

a) If there are ethical or legal restrictions on sharing a de-identified data set, please explain them in detail (e.g., data contain potentially identifying or sensitive patient information, data are owned by a third-party organization, etc.) and who has imposed them (e.g., a Research Ethics Committee or Institutional Review Board, etc.). Please also provide contact information for a data access committee, ethics committee, or other institutional body to which data requests may be sent.

b) If there are no restrictions, please upload the minimal anonymized data set necessary to replicate your study findings to a stable, public repository and provide us with the relevant URLs, DOIs, or accession numbers. Please see http://www.bmj.com/content/340/bmj.c181.long for guidelines on how to de-identify and prepare clinical data for publication. For a list of recommended repositories, please see https://journals.plos.org/plosone/s/recommended-repositories. You also have the option of uploading the data as Supporting Information files, but we would recommend depositing data directly to a data repository if possible.

Please update your Data Availability statement in the submission form accordingly.

4. If the reviewer comments include a recommendation to cite specific previously published works, please review and evaluate these publications to determine whether they are relevant and should be cited. There is no requirement to cite these works unless the editor has indicated otherwise.

[Note: HTML markup is below. Please do not edit.]

Reviewers' comments:

Reviewer's Responses to Questions

**Comments to the Author**

1. Is the manuscript technically sound, and do the data support the conclusions?

Reviewer #1: Yes

Reviewer #2: Yes

Reviewer #3: Yes

**********

2. Has the statistical analysis been performed appropriately and rigorously?

Reviewer #1: Yes

Reviewer #2: Yes

Reviewer #3: Yes

**********

3. Have the authors made all data underlying the findings in their manuscript fully available?

The PLOS Data policy

Reviewer #1: Yes

Reviewer #2: Yes

Reviewer #3: Yes

**********

4. Is the manuscript presented in an intelligible fashion and written in standard English?

Reviewer #1: Yes

Reviewer #2: Yes

Reviewer #3: Yes

**********

Reviewer #1: This article assesses relationships between health status, HRQol and physical activity in relatively elderly patients with e-axSpA using a cross-sectional dataset.

This study revealed that health status and physical activity are closely correlated even in long-standing axial SpA patients.

Several points need to be clarified

1) Influence of lifestyle, daily physical activity, working status, economic status, educational background, etc., so-called social background.

2) Medication

3) If these factors affect health status, a statistical correction is needed.

Reviewer #2: This is a well-conducted and clearly written cross-sectional study based on a long-term r-axSpA cohort. The methodology is sound, with appropriate use of validated instruments (BASFI, BASMI, ASAS-HI, SF-36) and multivariable regression analyses. The handling of non-parametric data, assessment of collinearity, and model validation are all appropriate. The Discussion adequately addresses the limitations regarding potential selection bias and causal inference. Overall, the statistical and methodological quality is sufficient, and the findings are consistent and clinically meaningful.

Reviewer #3: Vigorous physical activity and physical function are strongly associated with better health in patients with long-standing radiographic axial spondyloarthritis (r-axSpA).

This cross-sectional cohort study (N=141), based on registry data obtained from a longitudinal (13-year follow-up) cohort study (N=210), The Long-term Outcome Ankylosing Spondylitis (LOAS)-study in western Sweden (Ref#19, 2012), aim to evaluate physical function, spinal mobility, muscle function, and physical activity in patients with r-axSpA compared to reference values from the general population.

This manuscript is well written, presented results of original research, performed analysis/statistics at a high standard, included study limitations, and provided a well-focused discussion with appropriate conclusion supported by the data. Although this manuscript is technically rigorous and meets the scientific and ethical standard for inclusion in PLOS One, its average level of importance would also be of interest for general rheumatology, physical therapy, or rehabilitation journals (e.g. Annals of Rheumatic Disease, where the authors had published their abstract entitled "Physical function and regular exercise is important for Health-related Quality of life in patients with radiographic axial Spondyloarthritis" in 2024).

Specific Comments to the Authors:

1. On page 6, Comparisons with reference values of health individuals, line 137, consider changing "for BASMI age-matched reference values within the third quartile (Q3)" to "for BASMI age-matched reference values within or less than the third quartile (Q3)" for clarity.

2. On page 9, Health status and HRQoL, line 198, consider changing "female participants reported poorer mental health than males (Table 2)" to "female participants reported poorer (lower scores <50) mental health than males (Table 2)".

3. On page 14, SF36-PCS, line 244-245, consider changing "In the total group, better leg strength and for the female subgroup, a higher amount of moderate physical activity were" to "In the total group (and male subgroup), better leg strength and for the female subgroup, a higher amount of moderate physical activity were" if that is correct.

4. On page 17, Discussion, line 307-308, please clarify why in the current cohort study vigorous physical activity is defined as >1 hour/week, whereas, in the original cohort study it is defined as >30 minutes 2-3 times/week. Are the criteria for defining vigorous physical activity for the two cohorts (N=141 vs N=210, respectively) same/comparable?

5. On page 23, Reference#19, used in reference to page 4, Participants, line 95-96, please clarify if it was Reference#19 or another article which used/defined the term "LOAS" (The Long-term Outcome Ankylosing Spondylitis study).

**********

what does this mean?). If published, this will include your full peer review and any attached files.

If you choose “no”, your identity will remain anonymous but your review may still be made public.

**Do you want your identity to be public for this peer review?**  For information about this choice, including consent withdrawal, please see our Privacy Policy

Reviewer #1: No

Reviewer #2: No

Reviewer #3: **Yes:**  Andrew L. Wong, MD

**********

[NOTE: If reviewer comments were submitted as an attachment file, they will be attached to this email and accessible via the submission site. Please log into your account, locate the manuscript record, and check for the action link "View Attachments". If this link does not appear, there are no attachment files.]

To ensure your figures meet our technical requirements, please review our figure guidelines: https://journals.plos.org/plosone/s/figures

You may also use PLOS’s free figure tool, NAAS, to help you prepare publication quality figures: https://journals.plos.org/plosone/s/figures#loc-tools-for-figure-preparation.

NAAS will assess whether your figures meet our technical requirements by comparing each figure against our figure specifications.

Attachment Submitted filename: CFeldthusen review4526.docx

# Author comment: Author response to Decision Letter 1

**DOI:** 10.1371/journal.pone.0360248.r002

16 Jun 2026

Many thanks to the editor and referees for positive response and valuable feedback. We have been taken all comments into consideration and revised the manuscript.

Here are our point by point response to the reviewers comments which has also been uploaded as a separate file.

Reviewer #1: This article assesses relationships between health status, HRQol and physical activity in relatively elderly patients with e-axSpA using a cross-sectional dataset.

This study revealed that health status and physical activity are closely correlated even in long-standing axial SpA patients.

Several points need to be clarified

1) Influence of lifestyle, daily physical activity, working status, economic status, educational background, etc., so-called social background.

Response: We agree that social background also may influence health status. The questionnaire about physical activity that the patients answered was an estimation of the mean physical activities during a regular week. This clarification has now been added in the manuscript in the description of the questionnaire. We therefore have no data on daily physical activity. Neither do we have any detailed information on the patients’ economic status. We do have information regarding working status and educational background which has now been added to table 1.

Since the patient group was rather old (mean age 60 years) and 31% of the patients were retired due to old age, the ability to make reliable statistical assumptions regarding the impact of working status is compromised. Though, we have now, added a variable about working status and retirement in table 1 to make this transparent.

Regarding educational status there were significant but weak correlations between educational status and both ASAS HI (rs= -0.250, p<0.001) and SF36-PCS (rs= 0.228, p<0.001). But educational status did not affect any of the models when entered in the regression model for the total group.

We found no significant difference in work status or educational status between sexes.

2) Medication

Response: Use of medication is presented in table 1. There was a significant correlation between daily use of NSAID and SF36-PCS (rs= -0.203, p<0.05), however negligible. No other significant correlations were found between medication and health status. Use of medication did not affect any of the models when entered in the regression model for the total group.

3) If these factors affect health status, a statistical correction is needed.

Response: We found no influence on health status regarding use of medication or educational status, thus no correction for medication or educational status was needed.

As approximately one third of the patients was retired due to old age and 75% of the rest of the patients were working full time, working status was not considered a reliable factor for the analysis but presented in table 1 to describe the population.

Reviewer #2: This is a well-conducted and clearly written cross-sectional study based on a long-term r-axSpA cohort. The methodology is sound, with appropriate use of validated instruments (BASFI, BASMI, ASAS-HI, SF-36) and multivariable regression analyses. The handling of non-parametric data, assessment of collinearity, and model validation are all appropriate. The Discussion adequately addresses the limitations regarding potential selection bias and causal inference. Overall, the statistical and methodological quality is sufficient, and the findings are consistent and clinically meaningful.

Response: Thank you for your positive comments.

Reviewer #3: Vigorous physical activity and physical function are strongly associated with better health in patients with long-standing radiographic axial spondyloarthritis (r-axSpA).

This cross-sectional cohort study (N=141), based on registry data obtained from a longitudinal (13-year follow-up) cohort study (N=210), The Long-term Outcome Ankylosing Spondylitis (LOAS)-study in western Sweden (Ref#19, 2012), aim to evaluate physical function, spinal mobility, muscle function, and physical activity in patients with r-axSpA compared to reference values from the general population.

This manuscript is well written, presented results of original research, performed analysis/statistics at a high standard, included study limitations, and provided a well-focused discussion with appropriate conclusion supported by the data. Although this manuscript is technically rigorous and meets the scientific and ethical standard for inclusion in PLOS One, its average level of importance would also be of interest for general rheumatology, physical therapy, or rehabilitation journals (e.g. Annals of Rheumatic Disease, where the authors had published their abstract entitled "Physical function and regular exercise is important for Health-related Quality of life in patients with radiographic axial Spondyloarthritis" in 2024).

Response: Thank you for the positive comments!

Specific Comments to the Authors:

1. On page 6, Comparisons with reference values of health individuals, line 137, consider changing "for BASMI age-matched reference values within the third quartile (Q3)" to "for BASMI age-matched reference values within or less than the third quartile (Q3)" for clarity.

Response: Thank you for this suggestion for clarity. We have changed according to your suggestion.

2. On page 9, Health status and HRQoL, line 198, consider changing "female participants reported poorer mental health than males (Table 2)" to "female participants reported poorer (lower scores <50) mental health than males (Table 2)".

Response: Thank you for this suggestion for clarity. We have changed according to your suggestion.

3. On page 14, SF36-PCS, line 244-245, consider changing "In the total group, better leg strength and for the female subgroup, a higher amount of moderate physical activity were" to "In the total group (and male subgroup), better leg strength and for the female subgroup, a higher amount of moderate physical activity were" if that is correct.

Response: Thank you for being observant. However, for SF36-PCS the p-value for leg strength (TST) for the male subgroup was 0.08. Our cut off was p<0.05, and even if 0.08 is borderline, we chose to consider leg strength as not significant in the male subgroup.

4. On page 17, Discussion, line 307-308, please clarify why in the current cohort study vigorous physical activity is defined as >1 hour/week, whereas, in the original cohort study it is defined as >30 minutes 2-3 times/week. Are the criteria for defining vigorous physical activity for the two cohorts (N=141 vs N=210, respectively) same/comparable?

Response: The two studies used different questionnaires with different response options for physical activity. That is the reason for the different definitions of vigorous physical activity in the different cohort studies. However, we find these definitions comparable.

5. On page 23, Reference#19, used in reference to page 4, Participants, line 95-96, please clarify if it was Reference#19 or another article which used/defined the term "LOAS" (The Long-term Outcome Ankylosing Spondylitis study).

Response: Yes, reference #19 is correct as it is the first published reference in which this long-term follow-up is described.

Attachment Submitted filename: Response to Reviewers.docx

# Peer review: Decision Letter 1

**DOI:** 10.1371/journal.pone.0360248.r003

**Authors:** Wesam Gouda

20 Jul 2026

Dear Dr. Feldthusen,

Thank you for submitting your manuscript to PLOS ONE. After careful consideration, we feel that it has merit but does not fully meet PLOS ONE’s publication criteria as it currently stands. Therefore, we invite you to submit a revised version of the manuscript that addresses the points raised during the review process.

publication criteria and not, for example, on novelty or perceived impact.

For Lab, Study and Registered Report Protocols: These article types are not expected to include results but may include pilot data.

==============================

Please submit your revised manuscript by Sep 03 2026 11:59PM. If you will need more time than this to complete your revisions, please reply to this message or contact the journal office at plosone@plos.org. When you're ready to submit your revision, log on to https://www.editorialmanager.com/pone/ and select the 'Submissions Needing Revision' folder to locate your manuscript file.

- A letter that responds to each point raised by the academic editor and reviewer(s). You should upload this letter as a separate file labeled 'Response to Reviewers'.
- A marked-up copy of your manuscript that highlights changes made to the original version. You should upload this as a separate file labeled 'Revised Manuscript with Track Changes'.
- An unmarked version of your revised paper without tracked changes. You should upload this as a separate file labeled 'Manuscript'.

If you would like to make changes to your financial disclosure, please include your updated statement in your cover letter. Guidelines for resubmitting your figure files are available below the reviewer comments at the end of this letter.

If applicable, we recommend that you deposit your laboratory protocols in protocols.io to enhance the reproducibility of your results. Protocols.io assigns your protocol its own identifier (DOI) so that it can be cited independently in the future. For instructions see: https://journals.plos.org/plosone/s/submission-guidelines#loc-laboratory-protocols. Additionally, PLOS ONE offers an option for publishing peer-reviewed Lab Protocol articles, which describe protocols hosted on protocols.io. Read more information on sharing protocols at https://plos.org/protocols?utm_medium=editorial-email&utm_source=authorletters&utm_campaign=protocols.

As the corresponding author, your ORCID iD is verified in the submission system and will appear in the published article. PLOS supports the use of ORCID, and we encourage all coauthors to register for an ORCID iD and use it as well. Please encourage your coauthors to verify their ORCID iD within the submission system before final acceptance, as unverified ORCID iDs will not appear in the published article. *Only*  the individual author can complete the verification step; PLOS staff *cannot*  verify ORCID iDs on behalf of authors.

We look forward to receiving your revised manuscript.

Kind regards,

Wesam Gouda, MD,PhD

Academic Editor

PLOS One

Journal Requirements:

1. If the reviewer comments include a recommendation to cite specific previously published works, please review and evaluate these publications to determine whether they are relevant and should be cited. There is no requirement to cite these works unless the editor has indicated otherwise.

2. Please review your reference list to ensure that it is complete and correct. If you have cited papers that have been retracted, please include the rationale for doing so in the manuscript text, or remove these references and replace them with relevant current references. Any changes to the reference list should be mentioned in the rebuttal letter that accompanies your revised manuscript. If you need to cite a retracted article, indicate the article’s retracted status in the References list and also include a citation and full reference for the retraction notice.

[Note: HTML markup is below. Please do not edit.]

Reviewers' comments:

Reviewer's Responses to Questions

**Comments to the Author**

Reviewer #1: All comments have been addressed

Reviewer #3: (No Response)

**********

2. Is the manuscript technically sound, and do the data support the conclusions?

Reviewer #1: Yes

Reviewer #3: Yes

**********

3. Has the statistical analysis been performed appropriately and rigorously?

Reviewer #1: Yes

Reviewer #3: Yes

**********

4. Have the authors made all data underlying the findings in their manuscript fully available?

The PLOS Data policy

Reviewer #1: Yes

Reviewer #3: Yes

**********

5. Is the manuscript presented in an intelligible fashion and written in standard English?

Reviewer #1: Yes

Reviewer #3: Yes

**********

Reviewer #1: Dear authors,

Thank you for your appropriate response.

You have a guarantee of good quality, enough to publish this article.

Reviewer #3: Vigorous physical activity and physical function are strongly associated with better health in patients with long-standing radiographi8c axial spondyloarthritis.

This cross-sectional cohort study (N=141) re-enrolled in 2022, was based on registry data/enrollment obtained from a longitudinal cohort study (with 13-year follow-up) initially started in 2009 (N=210), The Long-term Outcome Ankylosing Spondylitis (LOAS)- study in western Sweden (Ref #19, 2012), aimed to evaluate physical function, spinal mobility, muscle function, and physical activity in patients with r-axSpA compared to reference values from the general population.

Comments to the Author:

1. On page 4, under Participants, please clarify whether the term "The Long-term Outcome Ankylosing Spondylitis (LOAS)-study was first used to describe the initial cohort enrolled in 2009 (Ref #19) or for the present study cohort re-enrolled in 2022. Similarly, please clarify appropriate time utilization of this term "LOAS" in the supplementary Figure 1. Reference 19 (published in 2012) never used the term "LOAS". Thus, may also want to add in (under References) the first peer-reviewed publication that actually used and defined the term "LOAS".

2. On page 8, under Results, please clarify in the text whether the percent of patients on disease-modifying anti-rheumatic drugs (DMARDs) is correctly at 42%, as according to Table 1 it stated 17% were on csDMARD, 35% were on bDMARD, and 0.7% were on tsDMARD, totaling to 52% (or 52.7%) were on DMARDs.

3. On page 14, under SF36-PCS (and Table 4 on page 12), please clarify the statement "In the total group, better leg strength... were also associated with better physical health." According to Table 4, under SF36 PCS, since the TST p value for both Male (p=0.08) and Female (p=0.15) were nonsignificant, it is not clear to this reviewer how then can be p value for the TST Total group (Male + Female) can mathematically somehow become significant for better physical health/leg strength muscle function. Please recheck the data and p values in Table 4.

**********

what does this mean?). If published, this will include your full peer review and any attached files.

If you choose “no”, your identity will remain anonymous but your review may still be made public.

**Do you want your identity to be public for this peer review?**  For information about this choice, including consent withdrawal, please see our Privacy Policy

Reviewer #1: **Yes:**  Ichiro Yoshii, president of Yoshii Clinic, Shimanto City, 787-0033 Kochi Prefecture, Japan

Reviewer #3: No

**********

[NOTE: If reviewer comments were submitted as an attachment file, they will be attached to this email and accessible via the submission site. Please log into your account, locate the manuscript record, and check for the action link "View Attachments". If this link does not appear, there are no attachment files.]

To ensure your figures meet our technical requirements, please review our figure guidelines: https://journals.plos.org/plosone/s/figures

You may also use PLOS’s free figure tool, NAAS, to help you prepare publication quality figures: https://journals.plos.org/plosone/s/figures#loc-tools-for-figure-preparation.

NAAS will assess whether your figures meet our technical requirements by comparing each figure against our figure specifications.

Attachment Submitted filename: CFeldthusen Re-review71926.docx

# Author comment: Author response to Decision Letter 2

**DOI:** 10.1371/journal.pone.0360248.r004

28 Aug 2026

Response to reviewers

PONE-D-25-27651R1

Vigorous physical activity and physical function are strongly associated with better health in patients with long-standing radiographic axial spondyloarthritis.

Journal Requirements:

1. If the reviewer comments include a recommendation to cite specific previously published works, please review and evaluate these publications to determine whether they are relevant and should be cited. There is no requirement to cite these works unless the editor has indicated otherwise.

2. Please review your reference list to ensure that it is complete and correct. If you have cited papers that have been retracted, please include the rationale for doing so in the manuscript text, or remove these references and replace them with relevant current references. Any changes to the reference list should be mentioned in the rebuttal letter that accompanies your revised manuscript. If you need to cite a retracted article, indicate the article’s retracted status in the References list and also include a citation and full reference for the retraction notice.

Response: The reference list has been checked, and no references have been retracted.

There is an erratum for ref 3; Rudwaleit M et al 2009: The correction provides conflict of interest statements of individual authors which were not provided to the paper.

There is also an error published regarding ref 40; Haglund E et al 2012. The error is in a table. The authors state that the error does not affect the conclusion of the article.

[Note: HTML markup is below. Please do not edit.]

Reviewers' comments:

Reviewer's Responses to Questions

Comments to the Author

1. If the authors have adequately addressed your comments raised in a previous round of review and you feel that this manuscript is now acceptable for publication, you may indicate that here to bypass the “Comments to the Author” section, enter your conflict of interest statement in the “Confidential to Editor” section, and submit your "Accept" recommendation.

Reviewer #1: All comments have been addressed

Reviewer #3: (No Response)

________________________________________

2. Is the manuscript technically sound, and do the data support the conclusions?

The manuscript must describe a technically sound piece of scientific research with data that supports the conclusions. Experiments must have been conducted rigorously, with appropriate controls, replication, and sample sizes. The conclusions must be drawn appropriately based on the data presented.

Reviewer #1: Yes

Reviewer #3: Yes

________________________________________

3. Has the statistical analysis been performed appropriately and rigorously?

Reviewer #1: Yes

Reviewer #3: Yes

________________________________________

4. Have the authors made all data underlying the findings in their manuscript fully available?

The PLOS Data policy requires authors to make all data underlying the findings described in their manuscript fully available without restriction, with rare exception (please refer to the Data Availability Statement in the manuscript PDF file). The data should be provided as part of the manuscript or its supporting information, or deposited to a public repository. For example, in addition to summary statistics, the data points behind means, medians and variance measures should be available. If there are restrictions on publicly sharing data—e.g. participant privacy or use of data from a third party—those must be specified.

Reviewer #1: Yes

Reviewer #3: Yes

________________________________________

5. Is the manuscript presented in an intelligible fashion and written in standard English?

PLOS ONE does not copyedit accepted manuscripts, so the language in submitted articles must be clear, correct, and unambiguous. Any typographical or grammatical errors should be corrected at revision, so please note any specific errors here.

Reviewer #1: Yes

Reviewer #3: Yes

________________________________________

6. Review Comments to the Author

Please use the space provided to explain your answers to the questions above. You may also include additional comments for the author, including concerns about dual publication, research ethics, or publication ethics. (Please upload your review as an attachment if it exceeds 20,000 characters)

Reviewer #1: Dear authors,

Thank you for your appropriate response.

You have a guarantee of good quality, enough to publish this article.

Response: Thank you for this positive response

Reviewer #3: Vigorous physical activity and physical function are strongly associated with better health in patients with long-standing radiographi8c axial spondyloarthritis.

This cross-sectional cohort study (N=141) re-enrolled in 2022, was based on registry data/enrollment obtained from a longitudinal cohort study (with 13-year follow-up) initially started in 2009 (N=210), The Long-term Outcome Ankylosing Spondylitis (LOAS)- study in western Sweden (Ref #19, 2012), aimed to evaluate physical function, spinal mobility, muscle function, and physical activity in patients with r-axSpA compared to reference values from the general population.

Comments to the Author:

1. On page 4, under Participants, please clarify whether the term "The Long-term Outcome Ankylosing Spondylitis (LOAS)-study was first used to describe the initial cohort enrolled in 2009 (Ref #19) or for the present study cohort re-enrolled in 2022. Similarly, please clarify appropriate time utilization of this term "LOAS" in the supplementary Figure 1. Reference 19 (published in 2012) never used the term "LOAS". Thus, may also want to add in (under References) the first peer-reviewed publication that actually used and defined the term "LOAS".

Response: Thank you for pointing out this unclarity. It is true that the term LOAS-study was not used to describe the initial cohort but the 13-year follow-up cohort. We have clarified this in page 4 which now states:

“Patients in western Sweden were initially recruited to a cohort study in 2009 which has been previously described [19]. Inclusion criteria were diagnosis of r-axSpA according to the Modified New York Criteria (mNY) for ankylosing spondylitis (AS) [1] and age >18 years. Exclusion criteria were psoriasis, inflammatory bowel disease, dementia, pregnancy, and difficulties in understanding Swedish language. The patients enrolled in 2009 were subsequently invited to follow-up examinations after 5-years in 2014 and after 13-years in 2022-2023. The latter as part of the Long-term Outcome Ankylosing Spondylitis (LOAS)-study (ref PMID: 40660310). The present study focuses on the results from the 13-year follow-up in a cross-sectional manner. Out of originally 210 patients, 141 patients were included in the 13-year follow-up study from 05/01/2022 to 01/02/2023, see flowchart in supplementary Figure 1 (S1 Fig). “

We have added a reference that first defined the LOAS-study and revised the supplementary flowchart figure (S1 Fig) accordingly.

2. On page 8, under Results, please clarify in the text whether the percent of patients on disease-modifying anti-rheumatic drugs (DMARDs) is correctly at 42%, as according to Table 1 it stated 17% were on csDMARD, 35% were on bDMARD, and 0.7% were on tsDMARD, totaling to 52% (or 52.7%) were on DMARDs.

Response: The reason for this is that 15 patients were taking csDMARD in combination with bDMARD or tsDMARD. That is why the total score do not match. To clarify we have added this information as a footnote to the table.

3. On page 14, under SF36-PCS (and Table 4 on page 12), please clarify the statement "In the total group, better leg strength... were also associated with better physical health." According to Table 4, under SF36 PCS, since the TST p value for both Male (p=0.08) and Female (p=0.15) were nonsignificant, it is not clear to this reviewer how then can be p value for the TST Total group (Male + Female) can mathematically somehow become significant for better physical health/leg strength muscle function. Please recheck the data and p values in Table 4.

Response: Thank you for being observant. We have rechecked the data and all values are correct. The reason for the p value being significant for the total group and not the subgroups is the larger n which results in significance being achieved.

Attachment Submitted filename: Response to reviewers R2.docx

# Peer review: Decision Letter 2

**DOI:** 10.1371/journal.pone.0360248.r005

**Authors:** Wesam Gouda

7 Sep 2026

Dear Dr. Feldthusen,

Thank you for submitting your manuscript to PLOS One. After careful consideration, we feel that it has merit but does not fully meet PLOS One’s publication criteria as it currently stands. Therefore, we invite you to submit a revised version of the manuscript that addresses the points raised during the review process.

Please submit your revised manuscript by Oct 22 2026 11:59PM. If you will need more time than this to complete your revisions, please reply to this message or contact the journal office at plosone@plos.org. When you're ready to submit your revision, log on to https://www.editorialmanager.com/pone/ and select the 'Submissions Needing Revision' folder to locate your manuscript file.

- A letter that responds to each point raised by the academic editor and reviewer(s). You should upload this letter as a separate file labeled 'Response to Reviewers'.
- A marked-up copy of your manuscript that highlights changes made to the original version. You should upload this as a separate file labeled 'Revised Manuscript with Track Changes'.
- An unmarked version of your revised paper without tracked changes. You should upload this as a separate file labeled 'Manuscript'.

If you would like to make changes to your financial disclosure, please include your updated statement in your cover letter. Guidelines for resubmitting your figure files are available below the reviewer comments at the end of this letter.

If applicable, we recommend that you deposit your laboratory protocols in protocols.io to enhance the reproducibility of your results. Protocols.io assigns your protocol its own identifier (DOI) so that it can be cited independently in the future. For instructions see: https://journals.plos.org/plosone/s/submission-guidelines#loc-laboratory-protocols. Additionally, PLOS One offers an option for publishing peer-reviewed Lab Protocol articles, which describe protocols hosted on protocols.io. Read more information on sharing protocols at https://plos.org/protocols?utm_medium=editorial-email&utm_source=authorletters&utm_campaign=protocols.

As the corresponding author, your ORCID iD is verified in the submission system and will appear in the published article. PLOS supports the use of ORCID, and we encourage all coauthors to register for an ORCID iD and use it as well. Please encourage your coauthors to verify their ORCID iD within the submission system before final acceptance, as unverified ORCID iDs will not appear in the published article. *Only*  the individual author can complete the verification step; PLOS staff *cannot*  verify ORCID iDs on behalf of authors.

We look forward to receiving your revised manuscript.

Kind regards,

Wesam Gouda, MD,PhD

Academic Editor

PLOS One

Journal Requirements:

If the reviewer comments include a recommendation to cite specific previously published works, please review and evaluate these publications to determine whether they are relevant and should be cited. There is no requirement to cite these works unless the editor has indicated otherwise.

Please review your reference list to ensure that it is complete and correct. If you have cited papers that have been retracted, please include the rationale for doing so in the manuscript text, or remove these references and replace them with relevant current references. Any changes to the reference list should be mentioned in the rebuttal letter that accompanies your revised manuscript. If you need to cite a retracted article, indicate the article’s retracted status in the References list and also include a citation and full reference for the retraction notice.

[Note: HTML markup is below. Please do not edit.]

Reviewers' comments:

Reviewer's Responses to Questions

**Comments to the Author**

Reviewer #3: All comments have been addressed

**********

2. Is the manuscript technically sound, and do the data support the conclusions?

Reviewer #3: Yes

**********

3. Has the statistical analysis been performed appropriately and rigorously?

Reviewer #3: Yes

**********

4. Have the authors made all data underlying the findings in their manuscript fully available?

The PLOS Data policy

Reviewer #3: Yes

**********

5. Is the manuscript presented in an intelligible fashion and written in standard English?

Reviewer #3: Yes

**********

Reviewer #3: Minor comments to the Author:

1. On page 3, under Introduction, please clarify preference of "(EMMs); anterior uveitis," or "(EMMs), anterior uveitis,".

2. On page S1_Fig, please clarify preference of "un-bolded box" or "bolded box" for "Patients invited to 13-years follow-up in 2022, The LOAS-study N=180".

**********

what does this mean?). If published, this will include your full peer review and any attached files.

If you choose “no”, your identity will remain anonymous but your review may still be made public.

**Do you want your identity to be public for this peer review?**  For information about this choice, including consent withdrawal, please see our Privacy Policy

Reviewer #3: No

**********

[NOTE: If reviewer comments were submitted as an attachment file, they will be attached to this email and accessible via the submission site. Please log into your account, locate the manuscript record, and check for the action link "View Attachments". If this link does not appear, there are no attachment files.]

To ensure your figures meet our technical requirements, please review our figure guidelines: https://journals.plos.org/plosone/s/figures

You may also use PLOS’s free figure tool, NAAS, to help you prepare publication quality figures: https://journals.plos.org/plosone/s/figures#loc-tools-for-figure-preparation.

NAAS will assess whether your figures meet our technical requirements by comparing each figure against our figure specifications.

# Author comment: Author response to Decision Letter 3

**DOI:** 10.1371/journal.pone.0360248.r006

23 Sep 2026

Response to reviewer

Reviewer #3: Minor comments to the Author:

1. On page 3, under Introduction, please clarify preference of "(EMMs); anterior uveitis," or "(EMMs), anterior uveitis,".

Response: Thank you for this comment. The wording of the sentence has been improved to the following sentence. ”The disease is characterized by inflammation in the axial skeleton, enthesitis, and sometimes peripheral arthritis, as well as, to a varying degree, extra-musculoskeletal manifestations (EMMs): anterior uveitis, inflammatory bowel disease, and psoriasis”.

2. On page S1_Fig, please clarify preference of "un-bolded box" or "bolded box" for "Patients invited to 13-years follow-up in 2022, The LOAS-study N=180".

Response: The bolded boxes describe how many patients that were included at baseline, 5-years follow -up and 12-13 years follow-up, respectively. The un-bolded box describes the recruitment process and the number of patients invited for follow-up, this to make the process transparent. However, that box does not describe the patients included in the actual data collection. Therefor we prefer to keep that box un-bolded.

Attachment Submitted filename: Response to reviewers 3.docx

# Editor report: Decision Letter 3

**DOI:** 10.1371/journal.pone.0360248.r007

**Authors:** Wesam Gouda

24 Sep 2026

Vigorous physical activity and physical function are strongly associated with better health in patients with long-standing radiographic axial spondyloarthritis.

PONE-D-25-27651R3

Dear Dr. Feldthusen,

We’re pleased to inform you that your manuscript has been judged scientifically suitable for publication and will be formally accepted for publication once it meets all outstanding technical requirements.

Within one week, you’ll receive an e-mail detailing the required amendments. When these have been addressed, you’ll receive a formal acceptance letter and your manuscript will be scheduled for publication.

An invoice will be generated when your article is formally accepted. Please note, if your institution has a publishing partnership with PLOS and your article meets the relevant criteria, all or part of your publication costs will be covered. Please make sure your user information is up-to-date by logging into Editorial Manager at Editorial Manager® and clicking the ‘Update My Information' link at the top of the page. For questions related to billing, please contact billing support.

If your institution or institutions have a press office, please notify them about your upcoming paper to help maximize its impact. If they’ll be preparing press materials, please inform our press team as soon as possible -- no later than 48 hours after receiving the formal acceptance. Your manuscript will remain under strict press embargo until 2 pm Eastern Time on the date of publication. For more information, please contact onepress@plos.org.

Kind regards,

Wesam Gouda, MD,PhD

Academic Editor

PLOS One

Additional Editor Comments (optional):

Reviewers' comments:

# Editor report: Acceptance letter

**DOI:** 10.1371/journal.pone.0360248.r008

**Authors:** Wesam Gouda

PONE-D-25-27651R3

PLOS One

Dear Dr. Feldthusen,

I'm pleased to inform you that your manuscript has been deemed suitable for publication in PLOS One. Congratulations! Your manuscript is now being handed over to our production team.

At this stage, our production department will prepare your paper for publication. This includes ensuring the following:

* All references, tables, and figures are properly cited

* All relevant supporting information is included in the manuscript submission,

* There are no issues that prevent the paper from being properly typeset

You will receive further instructions from the production team, including instructions on how to review your proof when it is ready. Please keep in mind that we are working through a large volume of accepted articles, so please give us a few days to review your paper and let you know the next and final steps.

Lastly, if your institution or institutions have a press office, please let them know about your upcoming paper now to help maximize its impact. If they'll be preparing press materials, please inform our press team within the next 48 hours. Your manuscript will remain under strict press embargo until 2 pm Eastern Time on the date of publication. For more information, please contact onepress@plos.org.

You will receive an invoice from PLOS for your publication fee after your manuscript has reached the completed accept phase. If you receive an email requesting payment before acceptance or for any other service, this may be a phishing scheme. Learn how to identify phishing emails and protect your accounts at https://explore.plos.org/phishing.

If we can help with anything else, please email us at customercare@plos.org.

Thank you for submitting your work to PLOS One and supporting open access.

Kind regards,

PLOS One Editorial Office Staff

on behalf of

Dr. Wesam Gouda

Academic Editor

PLOS One
